# Characterization and isolation of a T-DNA tagged banana promoter active during *in vitro *culture and low temperature stress

**DOI:** 10.1186/1471-2229-9-77

**Published:** 2009-06-24

**Authors:** Efrén Santos, Serge Remy, Els Thiry, Saskia Windelinckx, Rony Swennen, László Sági

**Affiliations:** 1 Laboratory of Tropical Crop Improvement, Division of Crop Biotechnics, Katholieke Universiteit Leuven, Kasteelpark Arenberg 13, B-3001 Leuven, Belgium; 2 Current address: Centro de Investigaciones Biotecnológicas del Ecuador, Escuela Superior Politécnica del Litoral (ESPOL), Campus Gustavo Galindo, Km. 30.5 vía Perimetral, Apartado 09-01-5863, Guayaquil, Ecuador

## Abstract

**Background:**

Next-generation transgenic plants will require a more precise regulation of transgene expression, preferably under the control of native promoters. A genome-wide T-DNA tagging strategy was therefore performed for the identification and characterization of novel banana promoters. Embryogenic cell suspensions of a plantain-type banana were transformed with a promoterless, codon-optimized luciferase (*luc*^+^) gene and low temperature-responsive luciferase activation was monitored in real time.

**Results:**

Around 16,000 transgenic cell colonies were screened for baseline luciferase activity at room temperature 2 months after transformation. After discarding positive colonies, cultures were re-screened in real-time at 26°C followed by a gradual decrease to 8°C. The baseline activation frequency was 0.98%, while the frequency of low temperature-responsive luciferase activity was 0.61% in the same population of cell cultures. Transgenic colonies with luciferase activity responsive to low temperature were regenerated to plantlets and luciferase expression patterns monitored during different regeneration stages. Twenty four banana DNA sequences flanking the right T-DNA borders in seven independent lines were cloned *via *PCR walking. RT-PCR analysis in one line containing five inserts allowed the identification of the sequence that had activated luciferase expression under low temperature stress in a developmentally regulated manner. This activating sequence was fused to the *uidA *reporter gene and back-transformed into a commercial dessert banana cultivar, in which its original expression pattern was confirmed.

**Conclusion:**

This promoter tagging and real-time screening platform proved valuable for the identification of novel promoters and genes in banana and for monitoring expression patterns throughout *in vitro *development and low temperature treatment. Combination of PCR walking techniques was efficient for the isolation of candidate promoters even in a multicopy T-DNA line. Qualitative and quantitative GUS expression analyses of one tagged promoter in a commercial cultivar demonstrated a reproducible promoter activity pattern during *in vitro *culture. Thus, this promoter could be used during *in vitro *selection and generation of commercial transgenic plants.

## Background

The new generations of transgenic plants require more precisely regulated expression of transferred genes, which calls for the identification and characterization of novel promoters in higher plants. Species-specific promoters can be utilized for more precise dissections of basic biological processes as well as for the generation of transgenic crops with possibly more favourable public acceptance [1].

Characterization of plant genes *via *T-DNA tagging represents a powerful approach to uncover new regulatory sequences [2,3]. Promoter tagging makes use of a promoterless selectable or reporter gene flanking a T-DNA border. After integration into the plant genome, this reporter gene is activated by flanking promoter sequences thus enabling study of native expression patterns within original genomic contexts. Use of the luciferase (*luc*) gene as reporter gene allows real-time detection of the luciferase (LUC) enzyme in a non-invasive and non-destructive manner combined with high sensitivity [4]. Furthermore, the short half-life of LUC activity [5] allows the monitoring of dynamic gene expression changes, which makes the *luc *reporter gene ideal for tagging promoters and genes exhibiting induced or developmentally regulated expression.

However, to date, relatively few research groups have exploited the LUC reporter system for this purpose. Only recently, two gene-trap vectors containing the wild type *luc *gene were constructed and successfully used in the model plant *Arabidopsis thaliana *for identification of genes activated by light during seedling development [6]. Tagging of low temperature (LT) (6 to 8 h at 4°C), responsive promoters was also reported in *Arabidopsis *seedlings using a large-scale *in vivo *LUC screening system [7], but quantitative data on the level of induction or repression during or after LT treatment and on the developmental regulation of these responses were not presented. Most plant T-DNA tagging vectors have so far been designed with the *uidA *(β-glucuronidase) reporter gene, which excludes non-destructive and real-time activity screening of the gene(s) tagged [8]. In relation to tagging temperature-responsive genes, Mandal *et al*. [9] reported the identification of one (out of 1200 lines tested) tagged *Arabidopsis *line exhibiting β-glucuronidase (GUS) activity after a 16 h treatment at 4°C. Screening for tagged LT-responsive genes was recently also performed in rice by subjecting plant samples to LT before measuring GUS activity at room temperature [10].

To date, and to the best of our knowledge, no plant promoter showing specific inducible activity during *in vitro *culture has been isolated and utilized. Promoters with high and/or specific *in vitro *activity could be employed for multiple purposes: (i) modeling at a test-tube scale important traits and processes such as organ formation (e.g. root or flower induction), (ii) systematic comparison of *in vitro *regeneration *vs. in vivo *development, (iii) understanding genomic adaptation processes (e.g. somaclonal variation) during *in vitro *culture, (iv) discovering novel genes such as transcription factors that regulate the expression of specific genes important during the *in vitro *stage, and (v) limiting expression of selectable marker genes for generation of transgenic crops.

Bananas (*Musa *spp.) are the most important fruit crop on Earth but their genetic improvement is seriously hampered by high degrees of sterility in most edible, triploid cultivars [11]. Therefore, integration of biotechnological tools into banana improvement programs appears imperative, including generation of transgenic plants with useful traits added. Though several heterologous promoters have been used for genetic transformation of banana [12-16], no native promoter has been utilized so far to drive the expression of agronomically interesting genes. A high-throughput LUC tagging platform developed for banana [17] was therefore applied here for the identification and characterization of native promoters regulated during *in vitro *culture and/or by LT (8°C) treatment. At this temperature, in the field as well as under controlled conditions [18] banana growth is arrested in the interior of the pseudostem [19], and chilling injury occurs [20]. This temperature is regularly reached in subtropical production areas [21], and presents a real production constraint. A LT-reactive or -inducible promoter can thus be instrumental in designing a protection strategy, where a stress resistance gene would be switched on only when a stressing temperature is reached.

We performed a large-scale screening for LUC activation on T-DNA tagged cell cultures subjected to LT stress in different developmental stages of plant regeneration *in vitro*. Flanking regions were isolated from a selected line containing five inserted tags and characterized in detail. One upstream sequence was shown to exert promoter activity to LT stress in a developmentally regulated manner, *i.e*. induction at undifferentiated stage and during early differentiation but not in *in vitro *plants. The observed activity of this promoter was confirmed by its similar expression pattern after transfer to a different genetic background suggesting that it could be used reliably for transgenic applications.

## Results

### Tagging of promoters active during *in vitro *regeneration

**Figure 1:**
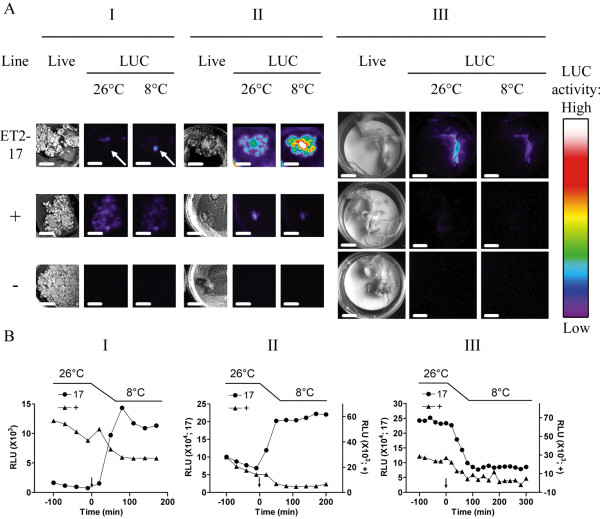
**Luciferase (LUC) activity at two temperatures in candidate promoter tagged line ET2-17 of 'Three Hand Planty' throughout *in vitro *regeneration**. (A) Representative images were taken under normal light (Live) and dark (LUC) conditions of candidate tagged line 17 transformed with pETKUL2 (promoterless *luc*^+ ^gene), a positive control line carrying the *luc*^+ ^gene under control of the enhanced CaMV 35S RNA promoter (+), and a negative untransformed control line (-). Developmental stages I, II, and III correspond to cell colony, differentiation and plantlet stage, respectively. The last LUC image recorded at 26°C and the LUC image recorded after 2 h at 8°C are depicted in pseudocolors (see color bar) with an upper greyscale limit setting of 1000. Arrows indicate the corresponding cell colony. Scale bars represent 1 cm. (B) Time course of LUC activity during temperature shift in promoter tagged line ET2-17 (17) and a positive (CaMV35S) transgenic control line (+) of banana throughout the *in vitro *regeneration process. LUC activity was monitored for 2 h at 26°C, then at time point zero (indicated by an arrow) temperature was set to 8°C, which was reached 1 h later, and then maintained for 2 h (stages I and II) or 4 h (stage III) (solid line above the graphs). The region of interest for quantification of LUC activity was standardized to 0.34, 0.58, and 23.19 cm^2 ^for stages I, II, and III, respectively. The Y axis scale are different for line 17 and the control line in stages II and III and indicated on either side of the graph

In order to search for endogenous promoters, banana cell suspensions were transformed with the promoter trap vector pETKUL2 (see Methods) carrying a promoterless luciferase gene. Two months after *Agrobacterium *transformation, screening of 15,887 independent cell colonies at room temperature revealed 155 (0.98%) cell colonies showing baseline activation (BLA). This result is comparable to BLA frequencies previously obtained with this construct (up to 2.5%, [17]). After discarding colonies exhibiting LUC activity, the remaining cell colonies were re-screened for BLA (26°C) one month later during 2 h followed by a LT treatment of 8°C while monitoring LUC activity in real-time for up to 10 h. This type of screening at cell colony stage (I) was repeated at the differentiation stage (II) when shoot induction is initiated and at the plantlet stage (III) for 10 responsive lines, as summarized (Table 1). Despite the early removal of positive colonies, BLA (26°C) was still detected at developmental stage I in all tagged lines (except for line 42) though at variable levels. LUC activity was absent at all times in untransformed control lines (Figure 1A) indicating that promoter sequences were tagged in these pETKUL2 transformed lines. The level of BLA varied greatly throughout *in vitro *regeneration for almost all tagged lines. For example, in tagged line 34, LUC activity at 26°C reached very strong, strong and moderate levels at developmental stages I, II and III, respectively (Table 1). In contrast, BLA remained very strong in line 156 throughout all stages of *in vitro *regeneration. Upon LT treatment (8°C) at stage I, enhanced LUC activity was observed in lines 17 and 42, which was lost in subsequent screenings at stage III and II, respectively (Table 1). The LUC activity pattern was monitored throughout *in vitro *regeneration as shown for line 17 in detail (Figure. 1A). The apparent lack of LUC activity at stage I at 26°C (Table 1) is due to the upper greyscale setting of 1000 in the LUC images. Comparison of LUC images suggests that the strongest up-regulation of LUC activity occurred at undifferentiated stage I in this line. The real-time observations strongly indicate a developmental regulation of the tagged promoter(s) in this line. In contrast, all other lines, including the positive control line (Figure 1A), showed a consistent decrease in LUC activity throughout *in vitro *regeneration in all tissueswhen subjected to LT (Table 1).

**Table 1:** Luciferase (LUC) activation during three developmental stages in 10 promoter tagged lines of 'Three Hand Planty'

	Developmental stage					
	
	I^a^		II^b^		III^c^	
			
Line	26°C	8°C	26°C	8°C	26°C	8°C
17	W	↑	M	↑	M	↓
42	N	↑	M	↓	W	↓
156	VS	↓	VS	↓	VS	↓
34	VS	↓	S	↓	M	↓
49	S	↓	S	↓	VS	↓
85	W	↓	VS	↓	S	↓
179	W	↓	VS	↓	S	↓
111	W	↓	W	↓	VS	↓
62	W	↓	W	↓	N	N
37	W	↓	W	↓	N	N
+^d^	M	↓	M	↓	W	↓

**Figure 2:**
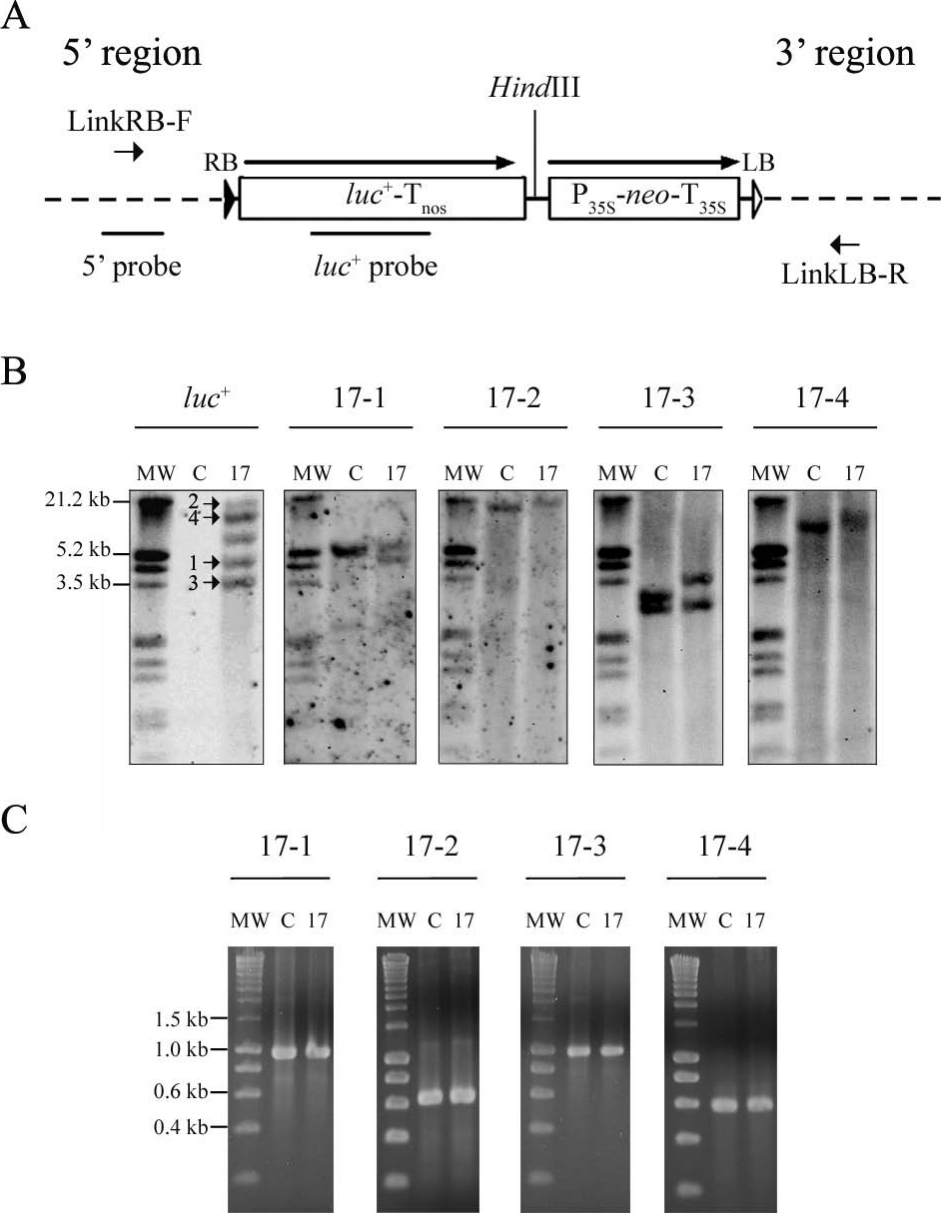
**Molecular characterization of tagged line ET2-17**. (A) Schematic representation of probe (thick lines) and primer (short arrows) positions for the different tagged sequences in line 17 transformed with promoter tagging vector pETKUL2. The position of the codon-optimized luciferase (*luc*^+^) and *neo *gene cassettes are shown with respect to the right (closed triangle) and left (open triangle) T-DNA border. Long arrows mark the direction of transcription. Dotted lines represent plant genomic DNA flanking the right (RB) and left (LB) T-DNA border, denominated 5' and 3' region, respectively. The drawing is not precisely according to scale. (B) Southern hybridization analysis of the *luc*^+ ^gene and the cloned 5' regions. Ten micrograms of total DNA were digested with *Hind*III, separated fragments were hybridized with a DIG-labeled *luc*^+ ^probe (862 bp) and rehybridized with a 5'-tagged sequence-specific probe (seq. 17-1: 422 bp, seq. 17-2: 425 bp, seq. 17-3: 435 bp and seq. 17-4: 165 bp). C: untransformed control plant. 17: tagged line ET2-17. MW: DIG-labeled DNA molecular marker III (Roche). The tagged *luc*^+ ^inserts are marked by arrows. (C) PCR confirmation in line ET2-17 of continuous tagged sequences with primers specific for 5'-tagged sequences (17-LinkRB-1F, 17-LinkRB-2F, 17-LinkRB-3F and 17-RT-4 for seq. 1–4, respectively) in combination with reverse primers specific for 3'-tagged sequences (17-LinkLB-1R, 17-LinkLB-2R, 17-LinkLB-3R and 17-LinkLB-4R). MW: SmartLadder (Eurogentec, Seraing, Belgium).

Based on the direct correlation between the number of greyscale levels as detected by the CCD camera and the level of LUC activity, lines were ranked at 26°C in five different classes: Not detectable (N), weak (W), moderate (M), strong (S) and very strong (VS). At each developmental stage real-time monitoring of LUC activity took place while subjecting to LT at 8°C (see Figure 1B) and 2 h later LUC activity was scored again. Upward and downward-pointing arrows indicate an increase and decrease, respectively, relative to the LUC activity at 26°C.

LUC activity detected according to upper greyscale limit setting: not detectable (N) – less than 500, weak (W) – between 500 and 3000, moderate (M) – between 3000 and 5000, strong (S) – between 5000 and 10,000, very strong (VS) – more than 10,000.

^a^Cell colony stage on ZZ medium 3 months after transformation.

^b^Differentiation stage on RD2 medium 8 months after transformation.

^c^*In vitro *regenerated plantlet on REG medium 15 months after transformation.

^d^Line transformed with positive control vector pETKUL3 [*luc*^+ ^gene fused to the enhanced Cauliflower Mosaic Virus (CaMV) 35S RNA promoter].

More detailed quantitative time course analyses of LUC activity were performed for tagged line 17 and the positive control line (Figure 1B). Calculation of the average LUC activity at 8°C and 26°C (excluding the images acquired during the transition phase) indicated for line 17 a 10.7-fold and a 2.5-fold increase in LUC activity when lowering the temperature at stage I and II, respectively and a 2.8-fold decrease in LUC activity at stage III (Table 1 and Figure 1B). In conclusion, as line 17 became more differentiated, less induction of LUC activity occurred upon LT stress indicating developmental regulation of LUC activity. In all screenings, LUC activity reached a new steady-state level within two hours following the change in temperature, which suggests a very fast response of the tagged promoters to LT and demonstrates the suitability of the LUC reporter gene system for kinetic real-time *in planta *studies in banana.

### Molecular characterization of tagged sequences

The number of T-DNA fragments integrated in seven of the promoter tagged lines was determined by Southern hybridization with a *luc*^+^-specific probe (Figure 2A) and ranged from 1 to 5 with an average of 3.3 T-DNA inserts per line (Table 2). To increase the success rate of isolating all 5'-tagged T-DNA flanking sequences in multicopy lines, both thermal asymmetric interlaced PCR (TAIL-PCR) with three degenerate primers and inverse PCR (I-PCR) with two restriction enzymes were performed. In TAIL-PCR, degenerate primer AD2-5 yielded less 5'-tagged sequences than the other two degenerate primers (1.6 *vs*. 2.3 and 2.9, respectively), while in I-PCR more 5'-tagged flanking sequences were obtained with *Bcl*I than with *BsrG*I (3.0 *vs*. 2.0, respectively). Although usually different tagged sequences were obtained with the two walking methods, identical sequences were also retrieved in several lines (data not shown), including tagged line 17 (Table 2). The number of isolated 5'-tagged sequences corresponded well (except for lines 156 and 49) with the number of T-DNA insertions (Table 2). Sequence analysis revealed that one T-DNA fragment was rearranged from the selectable marker gene cassette upstream to the *luc*^+ ^gene in lines 156, 49 and 111. In addition, T-DNA tandem repeats were identified in lines 156 and 49, and vector backbone sequences were integrated in lines 17, 156, 49 and 179 (data not shown). Despite these complex T-DNA rearrangements, BLA in these lines either remained stable or even increased throughout *in vitro *regeneration (Table 1, Figure 1).

**Table 2:** Number of T-DNA copies and 5'-tagged sequences in 'Three Hand Planty' lines transformed with promoter tagging vector pETKUL2

		No. 5'-tagged sequences					
		
		TAIL-PCR			I-PCR		Different sequences^a^
			
ET2-Line	T-DNA copies	AD2	AD2-1	AD2-5	*Bsr*GI	*Bcl*I	
17	5	3	5	2	2	2	5
156	5	2	1	2	2	2	3
49	4	4	5	1	NT	4	6
179	4	2	3	3	3	5	4
85	3	2	1	1	2	NT	3
111	3	2	3	1	1	3	2
34	1	1	2	1	2	2	1
Average	3.3	2.3	2.9	1.6	2.0	3.0	3.4

The minimum number of T-DNA copies was determined by Southern hybridization with a DIG-labelled *luc*^+ ^probe. Isolation of 5'-tagged sequences was accomplished using three different arbitrary degenerated (AD) primers in TAIL-PCR and two different restriction enzymes in I-PCR.

^a^Number of different 5'-tagged sequences obtained.

NT, not tested.

Due to the relative strength and developmentally (up)regulated pattern of LUC activity under LT stress (Table 1, Figure 1), line 17 was chosen for further molecular analysis. Southern hybridizations demonstrated five *luc*^+ ^inserts in line 17 (Table 2 and Figure 2B). A comparison between the hybridization patterns obtained with the *luc*^+ ^probe and the 5'-tagged sequence-specific probes (indicated as 5' probe on Figure 2A and with numbers 1 to 4 above the blots in Figure 2B) on the same blots revealed common fragments. For four of five *luc*^+ ^inserts in line 17, physical linkage with a cloned 5' region was established as shown in Figure 2B. The fifth 5'-tagged region contained a vector backbone rearrangement and could not be linked to the remaining *luc*^+ ^insert. For additional characterization, 3'-tagged regions were isolated with TAIL- and I-PCR either downstream of the left border in tagged line 17 and/or from cloned 5' sequences in untransformed plants. To find out which 3'-tagged sequence formed a continuous sequence with a cloned 5' region, 'linking' PCR was performed for each 5' region with all 3' sequence-specific primers (LinkRB-F and LinkLB-R in Figure 2A, respectively). Specific products with the calculated length were obtained both in untransformed control plants and in tagged plants of line 17 for four sequences in line 17 (17-1 to 17-4), which indicates sequence continuity between the corresponding isolated 5' and 3' regions (Figure 2C). Since tagged line 17 is hemizygous for the tags, the presence of PCR products with the same size as in the untransformed plant indicates amplification of the wild-type gene copies in tagged line 17. Amplification from the tagged loci was not expected under the PCR conditions applied as these contain (at least) the whole T-DNA (4463 bp) of pETKUL2 inserted, Specificity of all PCR products in Figure 2C was confirmed by nucleotide sequencing (data not shown).

These results demonstrate that up- and downstream tagged sequences can be retrieved and linked in banana irrespective of the T-DNA copy number. This is of primary importance when dealing with an interesting transgenic line because between 30% [22] and 85% (Table 2) of transgenic banana lines may contain more than one T-DNA copy.

### Sequence and RT-PCR analysis of tagged regions in line 17

**Figure 3:**
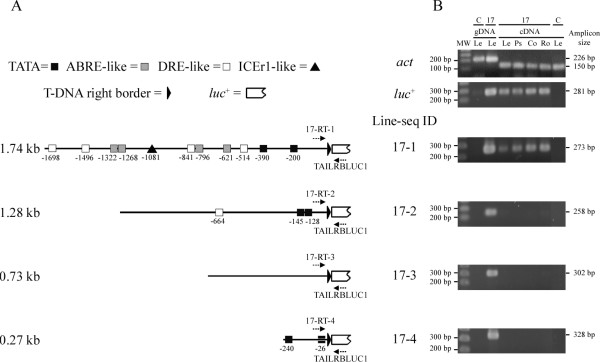
***Cis*-acting elements present in the 5'-tagged regions of tagged line ET2-17 and RT-PCR analysis of their regulation of the *luc*^+ ^gene**. (A) The presence of putative TATA boxes, ABRE-, and DRE-like *cis*-acting elements in the 5'-tagged sequences as determined by querying the PlantCARE and PLACE databases. The core sequence of the ICEr1 *cis*-acting element (CACATG) was manually located in sequence 17-1. Positions refer to the distance from the RB junction (bps). Primers used for RT-PCR are illustrated with dotted arrows. Drawings of 5' regions are according to scale. (B) RT-PCR analysis in different *in vitro *plantlet tissues at room temperature for the housekeeping actin gene (*act*), the codon-optimized luciferase gene (*luc*^+^) and the different 5'-tagged sequences performed at 35 amplification cycles. Actin primers flank an intron allowing discrimination between a genomic DNA product (225 bp) and the cDNA product (150 bp). Transcription of the 5'-tagged sequences 17-1, 17-2, 17-3 and 17-4 of line 17 was verified employing a sequence-specific forward primer (17-RT-1, 17-RT-2, 17-RT-3 and 17-RT-4, respectively) each time in combination with the reverse primer TAILRBLUC1. C: untransformed control plant. 17: tagged line ET2-17. Le: leaf; Ps: pseudostem; Co: corm; Ro: root, all from *in vitro *plants. MW: SmartLadder SF (Eurogentec, Seraing, Belgium).

To search for the presence of promoter sequences within the tagged 5' regions, an *in silico *search for *cis*-acting elements was performed (Figure 3A). Indicated are the elements involved in drought and/or LT responses (dehydration-responsive element, DRE; induction of C-repeat/DRE-element binding factor (CBF) expression region 1, ICEr1) and abscisic acid responses (abscisic acid-responsive element, ABRE), as well as candidate TATA boxes. The 1.74 kb sequence 17-1 [GenBank: EU161097] contains four DRE-like, one ICEr1-like, and four ABRE elements, which is significantly more than any of the other 5'-tagged regions. Two candidate TATA boxes are located at positions -390 and -200 relative to the T-DNA right border junction with all other elements upstream of them. Additionally, a TATA-less promoter sequence was identified in 17-1 with a corresponding candidate transcription start site at position -516, as determined by the TSSP software. The lack of homology to any available sequence in the databases for this 5'-tagged sequence plus the corresponding linked 3' region suggests that a cryptic promoter might be tagged in sequence 17-1. Only one DRE-like element and two putative TATA boxes were located in the 1.28 kb sequence 17-2. Analysis of the corresponding 1.4 kb 3'-tagged sequence revealed high homology over a length of 281 bp to a 596-bp banana EST (6000092615T1; 96% identity at E < 1 × 10^-134^) indicating that the corresponding T-DNA insertion had occurred in a coding region. This region shows homology to the last 90 amino acids of an unknown rice protein [GenBank: BAD87356, 74% identity and 87% positives at E < 2e^-32^] and 85 amino acids of another unknown rice protein [GenBank: NP_916242, 71% identity and 87% positives at E < 2e^-29^]. The sequence of the specific 642 bp PCR product containing the linked 5' and 3' flanking regions (Figure 2C, sequence 17-2) confirmed that the two flanks form one continuous sequence in the banana genome, but no homology was found to any known sequence. With the exception of two candidate TATA boxes in sequence 17-4, no other relevant promoter elements were identified in the two remaining 5'-tagged sequences 17-3 and 17-4 (Figure 3A) and database searches did not reveal homology to any known sequence for these 5' regions and their linked 3' regions.

To find out which sequence(s) activated the *luc*^+ ^reporter gene in tagged line 17, an RT-PCR approach was followed. First, RT-PCR of the housekeeping *act *gene demonstrated the absence of genomic DNA in the cDNA preparations (Figure 3B). Second, transcription of the *luc*^+ ^gene clearly occurred in all *in vitro *plant tissues tested (Figure 3B), which is in agreement with the real-time measurements at 26°C and developmental stage III (Table 1, Figure 1). Third, RT-PCR reactions were performed with a primer binding within a distance of 50 to 70 bp from the RB T-DNA junction of each of the four 5'-tagged sequences in combination with a *luc*^+^-specific primer. A product of the expected size (273 bp) was obtained in all tissues tested but only for sequence 17-1 (Figure 3B), confirming that this sequence is transcriptionally fused to the activated *luc*^+ ^gene. The expected PCR signal of 258 bp was absent for sequence 17-2, but appeared when rising the number of RT-PCR cycles from 35 to 40 showing that 17-2 weakly activates the *luc*^+ ^gene as well. The identity of this RT-PCR product was also confirmed by hybridization with a 17-2 sequence specific probe (data not shown). Results from semi-quantitative RT-PCR on samples of proliferating material initiated from *in vitro *apical meristems of line 17 was consistent with these results (data not shown) indicating that sequences 17-1 and 17-2 are also transcriptionally active in proliferating meristem cultures.

**Figure 4:**
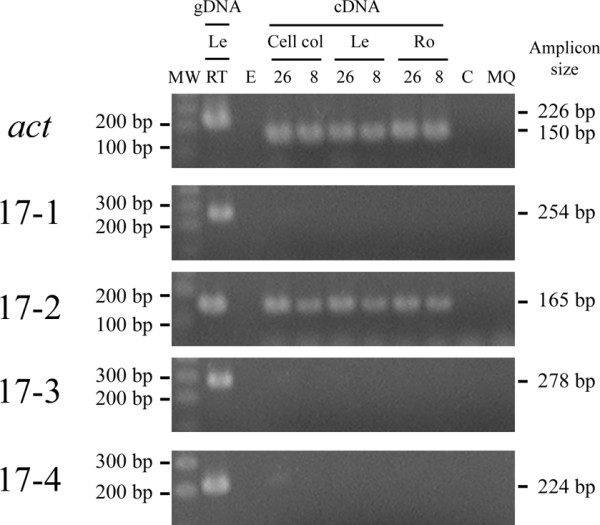
**RT-PCR analysis in untransformed tissues of the cultivar 'Three Hand Planty' at 26°C (26) and 8°C (8) treatments for the housekeeping actin gene (*act*) and the different 3'-tagged sequences of line 17**. Actin primers flank an intron allowing discrimination between a genomic DNA product (225 bp) and the cDNA product (150 bp). Transcription of the 3'-tagged sequences 17-1, 17-2, 17-3 and 17-4 of line 17 was verified employing the sequence-specific primer pairs 17-RTLB-1/17-LinkLB-1R, 17-RTLB-2/17LinkLB-2R, 17-RTLB-3/17-Link-3R and 17-RTLB-4/17-linkLB-4R, respectively. Cell col: cell colonies (developmental stage I). Le (leaf tissue) and Ro (root tissue) from *in vitro *plants (developmental stage III). RT: room temperature (25 ± 2°C). C: water control in cDNA synthesis. MQ: water control in the PCR reaction. PCR performed on RNA was negative for all the primer combinations (data not shown). MW: SmartLadder SF (Eurogentec, Seraing, Belgium).

We also investigated transcription of all 3' tagged sequences of line 17 by RT-PCR in cell colonies (stage I) as well as leaf and root tissues of *in vitro *plants (stage III) of untransformed control lines (Figure 4). Only the 3'-tagged sequence of insertion 17-2 was transcribed in the different tissues tested at 26°C and 8°C as shown by the expected RT-PCR signal of 165 bp (Figure 4), which corroborates the *in silico *analysis.

### Promoter activity of tagged sequence 17-1

**Figure 5:**
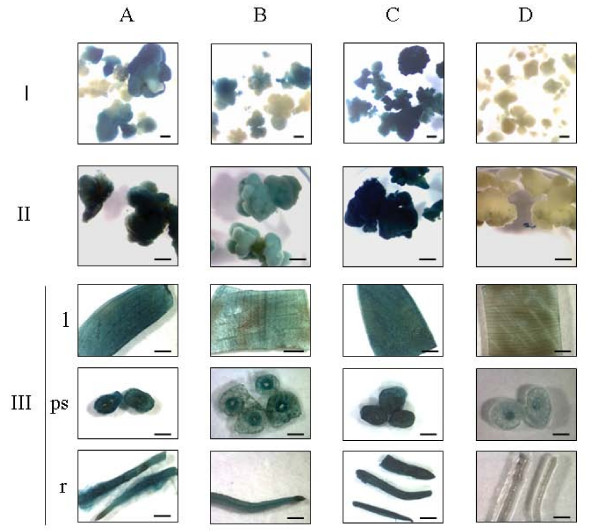
**Histochemical GUS assays throughout *in vitro *regeneration of transgenic dessert banana 'Grand Naine' after back-transformation with *uidA *gene fusions to tagged promoter sequence 17-1**. Developmental stages I, II, and III correspond to cell colony (2 month), differentiation (6 month) and *in vitro *plantlet (11 month) stage, respectively. Leaf (l), pseudostem (ps) and root (r) explants were tested at stage III. The *uidA *gene was put under the control of (A) the full-length promoter sequence 17-1 (1738 bp) (B) a 1350 bp deletion variant of promoter sequence 17-1 and (C) the maize ubiquitin promoter (positive control). (D) Negative untransformed control. Scale bars represent 1 mm.

To confirm promoter characteristics of tagged sequence 17-1, two transcriptional fusions (a full-length and a truncated one, see Methods) of sequence 17-1 to the *uidA *reporter gene were constructed and used for transformation of a commercial dessert banana variety. Transient GUS analysis of undifferentiated 'Grand Naine' suspension cells after 6 days of cocultivation revealed weak promoter activity of sequence 17-1 irrespective of its length compared to the positive control maize ubiquitin promoter (less than five *vs*. more than 500 blue spots per 50 mg fresh weight cells, respectively). Histochemical GUS analysis of transgenic cell colonies (stage I) at 26°C after two months of selection showed promoter activity of sequence 17-1 (Figure 5A), which confirms LUC activity measurements in the original tagged line 17 (stage I, Table 1 and Figure 1). Similar results were obtained with the 388 bp 3' truncation of sequence 17-1 (Figure 5B). As expected, the activity of these sequences appeared lower than that of the positive control maize ubiquitin promoter (Figure 5C). No background activity was detected in untransformed control cultures (Figure 5D) or cultures transformed with the empty control vector pCAMBIA-1391Z (data not shown).

**Figure 6:**
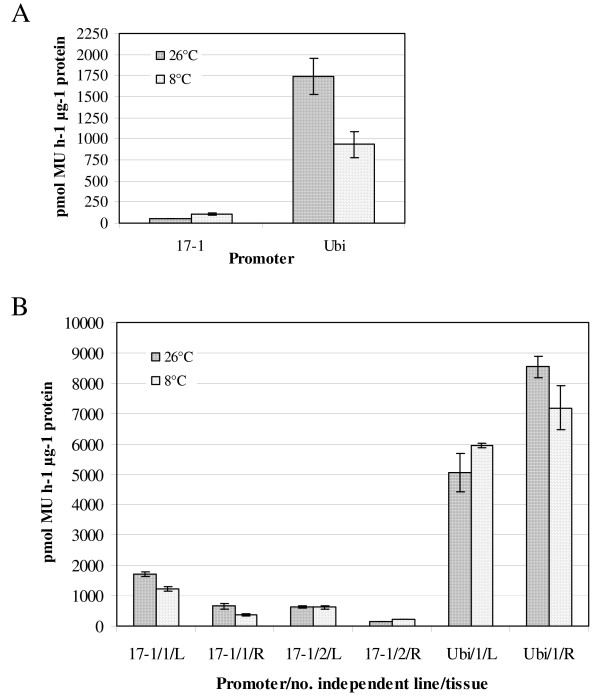
**GUS enzymatic activity in back-transformed lines of the dessert banana 'Grande Naine' carrying *uidA *gene fusion to the full-length promoter sequence 17-1 or the positive control maize ubiquitin promoter**. Each entry is the average ± standard error (± SE) result of three independent measurements after correction for the background obtained by untransformed controls. Cultures transformed with the empty control vector pCAMBIA-1391Z were not distinguishable from untransformed controls. (A) GUS enzymatic activity assay of LT treated (8°C for 10 h) transgenic (undifferentiated) cell colonies (stage I) 6 months after back-transformation. For each entry, at least 30 independent cell colonies (170 mg fresh weight in total) were pooled. The SE for the activity of promoter sequence 17-1 at 26°C was zero. (B) GUS enzymatic activity assay of LT treated (8°C for 18 h) transgenic *in vitro *plants (stage III), 19 months after transformation. For each independent line proteins were extracted from at least 250 mg leaf (L) and 200 mg root (R) tissue. The SE for the activity of promoter sequence 17-1 in root tissue of line no. 2 is not visible (only ± 5 and ± 3, at 26°C and 8°C, respectively).

LT responsiveness was evaluated by GUS enzyme activity assays in cultures back-transformed with the full-length 17-1 sequence in stage I (Figure 6A). Similarly to the original tagged line 17, a significant (220%) increase by LT treatment was observed in stage I cell colonies, whereas cell colonies transformed with the control ubiquitin promoter exerted decreased GUS expression (Figure 6A).

Activity of the 17-1 promoter sequence was further monitored throughout *in vitro *regeneration by histochemical GUS staining (Figure 5). Six months after transformation early differentiating stage II cultures transformed with the full-length 17-1 sequence and 3' truncated sequence 17-1 stained blue (Figure 5A and 5B, respectively) in agreement with the LUC activity detected at this stage in the original tagged line 17 (Figure 1). A higher staining intensity was obtained with the full-length 17-1 sequence although it was still less than that in cultures transformed with the positive control ubiquitin promoter (Figure 5C). No staining occurred in untransformed control cultures (Figure 5D) or pCAMBIA-1391Z transformed cultures (data not shown). Twelve months after transformation, in stage III *in vitro *plants, the full-length 17-1 promoter sequence (Figure 5A) and its deletion variant (Figure 5B) remained active in all tissues tested (leaf, pseudostem and root), with the full-length version still exhibiting higher activity. These activity patterns confirmed LUC screening results of the original tagged line 17 as well as the aforementioned RT-PCR results. Except for a slight coloration in pseudostem cross sections, no background staining was observed in untransformed control tissues (Figure 5D) and pCAMBIA-1391Z transformed tissues (data not shown).

In agreement with the real-time LUC activity measurements in the regenerated (stage III) original tagged line 17, GUS enzymatic activity decreased by LT treatment both in leaf and root tissue of back-transformed line carrying the full-length 17-1 promoter sequence in one back-transformed line (Figure 6B). In another line, however, the LT treatment did affect GUS activity in leaf tissue, while it caused a slight increase in GUS activity in root tissue (Figure 6B). A tissue dependent reaction of the maize ubiquitin promoter in the regenerated positive control line was also detected. More specifically, an upregulation of GUS activity occurred in leaf tissue, whereas in root tissue GUS activity was downregulated (Figure 6B). Similar to cell colony stage I, the full-length 17-1 promoter sequence was less active than the maize ubiquitin promoter in stage III regenerated plants at both temperatures and tissues tested.

## Discussion

A combination of the improved T-DNA tagging vector pETKUL2 [17] and detailed real-time monitoring of *in vitro *activated LUC expression has allowed us to tag banana promoters and monitor their activation during *in vitro *regeneration. Three months after *Agrobacterium *infection of embryogenic suspension cells, high-throughput screening for promoter activated lines was started at cell colony stage and repeated at subsequent *in vitro *regenerative stages. Both qualitative scoring and quantitative measurements using image analysis software revealed an enhanced LUC activity under LT (8°C) stress during early undifferentiated developmental stages in two (lines 17 and 42) out of 10 LT-responsive lines. This result demonstrates reliability of the simple and fast qualitative scoring of recorded images, and suggests developmental regulation of LT upregulation of the tagged promoters in these lines. The latter conclusion is further corroborated by recapitulation of the LT upregulated LUC activity profile in similar cell colony cultures that were re-initiated *via *proliferation [23] from apical meristems of *in vitro *multiplied plants of line 17 (data not shown). To the best of our knowledge, no comparable real-time monitoring of T-DNA tagged cultures from undifferentiated cells until *in vitro *plants have been performed so far. Usually, only a specific phase of plant development was investigated, such as embryo development [24], seedling development [6,25], flowering [26] or lateral root development [27]. Similarly, LT upregulation has previously been studied either at a specific stage of development [7] or in certain tissue explants [10] but not in real-time and *in planta *throughout the whole plant regeneration process.

Since it is known that light intensity of the LUC reaction measured in solution decreases upon lowering the temperature [28-30], we hypothesize that non-LT responsive promoters are tagged in banana lines that show a similar decreased *in vivo *LUC activity when exposed to identical temperature regimes. Developmentally regulated promoters might, however, still be tagged in these cases as indicated by variable LUC levels emitted at 26°C throughout regeneration (Table 1). On the other hand, the observed BLA profiles of tagged lines might also be the result of regulation by one or more tissue culture components rather than simply by the developmental program during *in vitro *regeneration. Bade *et al*. [31] addressed this issue in promoter tagged *Brassica napus *lines and found that 6 out of 20 tagged promoters with callus-specific activity were also auxin upregulated. Interpretation of such results, however, will be difficult in practice because other components of the different media may also influence gene expression.

T-DNA promoter and gene tagging studies in model plants usually yield an average of one to two T-DNA copies per transgenic line [32-34]. Southern analysis of seven promoter tagged banana lines showed an average of 3.3 T-DNA copies per line. Because this makes isolation and identification of the activating 5'-tagged region(s) laborious and time consuming, single T-DNA copy integrations are usually preferentially analyzed [35-38]. Because of the low incidence of single copy T-DNA insertions in banana tagged lines, we demonstrate here that activated insertion(s) can be identified in multicopy T-DNA mutants. The combination of I-PCR and TAIL-PCR yielded the expected number of flanking sequences in the majority of lines) highlighting the usefulness of PCR walking techniques with different principles of operation for retrieval of the flanking sequences in multicopy T-DNA lines.

*In silico *analysis of the four 5'-tagged candidate promoter sequences in line 17 suggested that two promoters had been tagged. One T-DNA insertion (17-1) tagged a cryptic promoter since transcription of the corresponding 3'-tagged sequence in untransformed control tissues was absent both at developmental stage I and stage III, while the 5'-tagged 17-1 sequence displayed promoter activity following back-transformation (see below). Tagging of cryptic promoters, i.e. regulatory elements that are inactive at their native genomic positions but become functional upon adjacent insertion of (trans)genes, is not uncommon in plants and some of them also appear tissue-specific [35,36,39-41]. High homology of part of the downstream sequence to a banana EST and the last 90 amino acids of an unknown rice protein suggested that the other T-DNA insertion (17-2) occurred in a coding region. RT-PCR using primers specific for the 3'-tagged sequence of this insertion to detect transcription in tissues of stage I and stage III untransformed lines confirmed this finding. Moreover, the lack of transcription of the 3'-tagged sequences of insertions 17-3 and 17-4 in stage I cell colonies at 25°C and 8°C also strongly suggests that their corresponding 5'-tagged sequences did not contribute to the LT upregulated LUC activity in the original tagged line 17. Finally, using RT-PCR analysis we confirmed that both 5'-tagged sequences 17-1 and 17-2 activate the *luc*^+ ^gene, albeit to a different level with sequence 17-1 being the most active. This opens the possibility that promoter 17-1 is responsible for LT upregulation in undifferentiated cell cultures, while baseline LUC activity might be caused by activity of promoter 17-2 (and perhaps promoter 17-1).

Histochemical GUS analyses of back-transformed lines demonstrated that sequence 17-1 possesses promoter activity throughout *in vitro *regeneration and in all banana tissues tested. Whether this promoter remains active in mature plants will be investigated. Since the maize ubiquitin promoter is highly active in banana [12,13,16,42], it was expected that the 17-1 promoter would be weaker than the ubiquitin promoter as revealed by differential staining intensity of GUS assays. The results also demonstrated that sequence 17-1, originally isolated from an African plantain-type banana, is also functional in the commercial dessert banana 'Grand Naine'. These results are encouraging in view of generating cisgenic plants. The full-length sequence 17-1 was more active than the 3' truncated sequence suggesting that the 388 bp 3' truncation harbours elements that are essential for promoter strength but not the core functional elements. On the other hand, no other functional elements were likely removed since the deletion did not affect the expression pattern of this promoter.

With a more than 2-fold increase in GUS activity after a 10 h treatment at 8°C the expression pattern of the original tagged line 17 under LT stress at cell colony stage I was recapitulated in the back-transformed lines carrying the full-length 17-1 promoter sequence. This level of induction at 8°C is lower than in the original tagged line 17 (10.7-fold) and might be caused by stability of the GUS enzyme (half-life of more than 60 h). In regenerated *in vitro *plants (stage III), the decrease in LUC expression observed in the original tagged line 17 under LT stress (8°C for 18 h) was also detected by GUS enzyme activity assays in one out of two back-transformed lines carrying the full-length 17-1 promoter sequence irrespective of the tissue tested. A position effect might contribute to the different activity pattern of the 17-1 sequence in the other back-transformed line. Nevertheless, these results support the aforementioned histochemical data that the full-length 17-1 sequence has promoter activity in all tissues tested at stage III. The almost 2-fold decrease in GUS activity in the positive control cell colonies under LT stress might be attributed to an inhibiting effect of LT on the maize ubiquitin promoter activity. A similar and consistent 2- to 4-fold lower GUS activity at 4°C than at 24°C in wheat leaves controlled by the maize ubiquitin promoter has been reported [43]. In contrast, exposure of two stably transformed (*UBI1:GUS*) rice callus lines to freezing (-20°C for 1 h) resulted in an approximately 3-fold increased GUS expression [44]. Moreover, in the control transgenic line tested at regenerated plant stage III in this work, the maize ubiquitin promoter showed an opposite reaction to LT stress in different tissues. LT conditions and tissue type may therefore play an important role in the induction or suppression of the maize ubiquitin promoter, which also warrants further investigation on the activation of promoter sequence 17-1 in different tissues under different LT conditions. The same putative *cis*-regulatory DRE and ABRE elements found in the maize ubiquitin promoter [44] and present in several LT-responsive promoters [45,46] were also identified in promoter sequence 17-1 and could contribute to increased expression observed during the LT treatment.

In summary, we have devised and applied a real-time *in vitro *screening platform for the identification of promoter-tagged cultures as well as for the isolation of an LT-responsive promoter in banana. This or a similar system can be employed in any plant species with a comparable *in vitro *regeneration procedure (e.g. embryogenic callus phase in most cereal species) because homogeneous cell or tissue cultures can be reliably and uniformly induced, for example, by temperature or a chemical treatment. The presented platform could be an important tool for screening other traits and finding genes that are induced by a broad range of factors, possibly including high temperature, oxidative stress (e.g. paraquat treatment), salt/osmotic or drought stress, as well as elicitors or toxins. By appropriate modifications in tagging constructs the same platform can also be useful in other functional genomics strategies such as activation tagging.

## Conclusion

We have demonstrated here that a high-throughput real-time luciferase-based screening system is valuable for tagging novel promoters and genes in banana and to follow their expression pattern throughout *in vitro *development and LT treatment. In addition, with the aid of combined PCR techniques we were able to identify and isolate the activated insertions in a multicopy T-DNA line. Histochemical GUS analyses of back-transformed cell cultures of a different, commercial cultivar demonstrated that the isolated sequence 17-1 possesses a reproducible promoter activity pattern. Thus, this promoter represents a useful tool for regulated *in vitro *expression of foreign genes and might be employed during generation of commercial transgenic plants.

## Methods

### Plant material, transformation and regeneration

Embryogenic cell suspensions (ECS) of the plantain-type banana (*Musa *spp.) cultivar 'Three Hand Planty' (AAB genomic group, International Transit Center accession number, ITC.0185) were cocultivated with the *Agrobacterium tumefaciens *strain EHA105 harboring the plasmid pETKUL2 or pETKUL3, which carry a promoterless *luc*^+ ^gene [47] or the *luc*^+ ^gene under the control of the enhanced Cauliflower Mosaic Virus (CaMV) 35S RNA promoter, respectively [17]. Transformation, selection and regeneration of transgenic lines were performed according to Remy *et al*. [17]. Briefly, after 3 months of selection on ZZ medium (half-strength MS medium, 5 μM 2,4-D and 1 μM zeatin) containing 50 mg l^-1 ^geneticin and 200 mg l^-1 ^timentin, transgenic cell colonies were subcultured to RD1 medium (half-strength MS medium, 100 mg l^-1 ^*myo*-inositol) and 4 months later to RD2 medium (half-strength MS medium, 10 μM benzyladenine) to induce embryos and shoots, respectively. Eleven months after transformation, differentiating cultures were subcultured on REG medium (MS medium, 1 μM benzyladenine and 1 μM indole acetic acid) for plant regeneration. All cultures were kept at 26 ± 2°C in the dark until shoot formation and then transferred to REG medium and kept under a 16 h photoperiod with a photosynthetic photon flux density of 50 μE m^-2 ^s^-1 ^provided by Cool White fluorescent lamps (TLD 58W/33, Philips, France).

### Luciferase detection

Screenings for LUC activity were performed in several phases during the regeneration process. Cell colonies were analyzed on ZZ medium by adding 300 μl of luciferin at a concentration of 0.1 mM in half-strength MS medium 1 h before screening. Application of luciferin in half-strength MS medium was done twice (20 h and 1 h before screening) to differentiating cultures on RD2 medium. Regenerated *in vitro *plantlets were placed horizontally on filter paper saturated with liquid half-strength MS medium and sprayed 19 h and 1 h before screening with 0.1 mM luciferin dissolved in MilliQ water.

Detection of LUC activity was performed with a liquid nitrogen-cooled slow-scan charge-couple device (CCD) camera (VERSARRAY™ 512 B LN, Roper Scientific, Vianen, The Netherlands) equipped with a light sensitive camera lens (Nikkor F 50 mm f/1.2, Nikon, Tokyo, Japan) as described [17]. Briefly, samples were placed in a light-tight box, and a live image was first captured as reference, then LUC images were recorded with an integration time of 20 min. Time lapses were taken at an interval of 30 min for screenings on ZZ and RD2 medium, and 20 min for screenings of *in vitro *plantlets maintained on REG medium. Acquired images were analyzed by scaling the number of different grey values, and scored as very strong (VS), strong (S), moderate (M), weak (W) and not detectable (N) at an upper greyscale limit setting to more than 10,000, between 5000 to 10,000, 3000 and 5000, 500 and 3000 and to less than 500, respectively. For quantitative analysis, images were processed with the METAMORPH^® ^5.0r3 software (Universal Imaging, Downington, PA, USA).

### Screenings during development and LT treatment

Cell colonies showing baseline LUC activity (BLA) at room temperature after two months of transformation were discarded. One month later, LUC activation patterns of the remaining cell colonies were monitored in real-time by incubating them on a temperature-controlled metal plate in the light-tight box. Temperature was regulated by a circulating water bath (1140S, VWR International Inc., San Diego, CA, USA) and calibrated with a wired digital thermometer (indoor/outdoor model No. EM899, Oregon Scientific, Portland, OR, USA) in order to record 26°C or 8°C within the Petri dishes. Cultures were subjected to 26°C for a period of 2 h, 3 h and 6 h for screening on ZZ medium (stage I, cell colonies 3 months after transformation), RD2 medium (stage II, differentiating cultures 8 months after transformation), and REG medium (stage III, *in vitro *plantlets 15 months after transformation), respectively. LUC activity was quantified during the last 2 h at 26°C as well as during transition to 8°C and its maintenance for 2 h at stage I and II or 4 h at stage III. Visual monitoring of LUC activity at 8°C continued for up to 10 h at all stages. LUC activity was expressed in relative light units (RLU) and corrected for the background measured in an untransformed control line. The region of interest for quantification of LUC activity was standardized at 0.34, 0.58, and 23.19 cm^2 ^at stage I, II, and III, respectively.

### Southern hybridization analysis

A modified protocol based on the methods of Dellaporta *et al*. [48] and Aljanabi and Martínez [49] was used for DNA isolation from leaf tissue. Southern hybridization was performed according to Remy *et al*. [17]. Briefly, 10 μg of total DNA were digested overnight with *Hin*dIII in a volume of 100 μl. Probes for the *luc*^+ ^gene and right border (RB) T-DNA flanking sequences were produced using the PCR DIG-DNA Labeling Mix (Roche, Vilvoorde, Belgium). The substrate CSPD^® ^(Roche) was applied to detect the signals by immuno-chemiluminescence according to the manufacturer's instructions. Images were captured with the CCD camera described above at exposure times of up to 20 min.

### Isolation and characterization of T-DNA flanking sequences

Isolation of T-DNA flanks was accomplished with Thermal Asymmetric Interlaced-PCR (TAIL-PCR) and Inverse PCR (I-PCR). TAIL-PCR was performed according to Liu *et al*. [50] and Remy *et al*. [17] with the following modifications: low-stringency annealing was done at 42°C for 1 min, high-stringency annealing at 64°C for 1 min, and elongation at 68°C for 3.5 min. Three different degenerated primers were employed independently: AD2 [50], AD2-1 and AD2-5 [51]. T-DNA specific primers for the isolation of RB T-DNA flanking sequences were TAILRBLUC1, TAILRBLUC2 and LucR3 for the primary, secondary, and tertiary TAIL-PCR reaction, respectively [17]. For the left border (LB), the corresponding primers were TAILLBpET2n1, TAILLBpET2n2 and TAILLBpET2n3. For I-PCR, 250 ng DNA was digested with 10 units of either *Bsr*GI or *Bcl*I. Digested DNA was self-ligated overnight and PCR was performed in a total volume of 50 μl using primers LucL2 and TAILRBLUC1 for the isolation of RB T-DNA flanking sequences. A touchdown PCR program was performed with an initial denaturation step of 95°C for 2 min and four steps of 3 cycles each consisting of 95°C for 15 s; 68°C, 66°C, 64°C and 62°C for 20 s for each step, respectively; and 68°C for 3.5 min followed by 30 cycles of 95°C for 15 s, 60°C for 20 s, and 68°C for 3.5 min, with a final elongation step of 68°C for 5 min. Nested amplification of primary products was done with primers LucL3 and TAILRBLUC2 or LucR3. Isolation of LB T-DNA flanking sequences was accomplished using primers TAILLBpET2n1 and LucR5 or Luc+R. For semi-nested amplification, primers TAILLBpET2n2 or TAILLBpET2n3 were also employed. All primer sequences are listed in Additional file 1.

### Reverse transcription (RT-)PCR analysis and 'linking' PCR

Total RNA was isolated using the RNEASY^® ^Plus Mini Kit (Qiagen, Hilden, Germany) from 60–80 mg of leaf, pseudostem, corm, and root tissue of *in vitro *regenerated plantlets maintained at room temperature. One microgram of RNA was used for cDNA synthesis with the REVERTAID™ H Minus First Strand cDNA Synthesis Kit (Fermentas, St. Leon-Rot, Germany) using an oligo(dT)_18 _primer or the *luc*^+ ^sequence-specific Luc+R primer to detect transcription of the housekeeping actin (*act*) gene [52] or the *luc*^+ ^transgene, respectively. The RT-PCR reaction followed the cycling program: initial denaturation at 95°C for 2 min, followed by 35 cycles of 95°C for 30 s, 60°C for 30 s, and 68°C for 30 s with a final elongation step of 68°C for 2 min. Forward primers specific to the 5'-tagged regions 1, 2, 3, and 4 of line 17 and annealing near the RB insertion junction were 17-RT-1, 17-RT-2, 17-RT-3, and 17-RT-4, respectively and were employed in combination with the reverse primer TAILRBLUC1 specific to the *luc*^+ ^gene. Control primers for the *act *gene and the *luc*^+ ^transgene were ActinF3/ActinR2 and LucL2/LucR5, respectively (Additional file 1). To confirm continuity of 5'- and 3'-tagged regions, PCR was performed with a specific forward and reverse primer, respectively. For sequence 1, 2, 3, and 4 of the multiple T-DNA copy line 17, the primer pairs were 17-LinkRB-1F/17-LinkLB-1R, 17-LinkRB-2F/17-LinkLB-2R, 17-LinkRB-3F/17-LinkLB-3R, and 17-RT-4/17-LinkLB-4R, respectively (Additional file 1). The PCR reaction was programmed as described above. To investigate transcription of the LB T-DNA flanking sequences identified in tagged line 17, RT-PCR was performed on cell colonies (stage I) and tissues (leaf and root) of regenerated *in vitro *plants (stage III) of untransformed control lines of the cultivar 'Three Hand Planty'. Cell colonies were pooled to reach 60–80 mg tissue. RNA isolation, cDNA synthesis and RT-PCR reaction were performed as described above. For the LB T-DNA flanking sequences 1, 2, 3, and 4 the primer pairs were 17-RTLB-1/17-LinkLB-1R, 17-RTLB-2/17-LinkLB-2R, 17-RTLB-3/17-LinkLB-3R, 17-RTLB-4/17-LinkLB-4R, respectively (Additional file 1).

### Sequence analysis

All PCR products were cloned in the pCR4-TOPO^® ^vector (Invitrogen, Merelbeke, Belgium) and commercially sequenced. Sequences were analyzed with BLASTn and BLASTx  programs against the GenBank database, and against a banana EST database donated by Syngenta to the Global *Musa *Genomics Consortium. The 5'-tagged putative promoter sequences were queried in PlantCARE (bioinformatics.psb.ugent.be/webtools/plantcare/html/) as well as PLACE  databases, and analyzed with the TSSP  software.

### Cloning and back-transformation of tagged sequences

A 1742 bp and a 1354 bp fragment containing the respective regions of -1746 bp to -5 bp and -1746 bp to -393 bp relative to the RB insertion site of sequence 17-1 in tagged line 17 were amplified using the primer pairs 17-1F1/17-1R1 and 17-1F1/17-1R2, respectively (Additional file 1). The Extensor High-Fidelity PCR Enzyme mix (ABgene, Epsom, UK) was used for reliable amplification and the amplicons were cloned into the pCR4-TOPO^® ^vector. Following digestion by *Eco*RI the putative promoter sequences were inserted into the *Eco*RI linearized pCAMBIA-1391Z vector, which contains a promoterless *uidA*^INT ^gene downstream of the multiple cloning site . As positive control, the 2053 bp promoter and first intron from the maize ubiquitin gene [53], one of the strongest known promoters in monocot plants, was inserted into pCAMBIA-1391Z. All constructs were verified by sequencing and transferred to *Agrobacterium tumefaciens *strain EHA105 by electroporation. Transformation of ECS of the commercial dessert banana cultivar 'Grand Naine' (AAA, ITC.1256) was performed as described [54]. After two to three months of selection on ZZ medium containing 50 mg l^-1 ^hygromycin and 200 mg l^-1 ^timentin, proliferating cell colonies maintained at 26 ± 2°C were histochemically stained or fluorometrically analyzed for GUS activity according to modified procedures of Jefferson *et al*. [55]. Staining was done in a X-Gluc solution containing 100 mM Tris-HCl (pH 8.0), 1% (w/v) 5-bromo-4-chloro-3-indolyl-β-glucuronic acid dissolved in DMSO, 0.5 mM ferrocyanide, 0.5 mM ferricyanide, 1% (w/v) ascorbic acid, 10 mM EDTA and 2% (w/v) CHAPS (3-[(3-cholamidopropyl)dimethylammonio]-1-propanesulfonate) detergent (Roche), while 20% (v/v) methanol was included in the fluorometric assay buffer. Early differentiating stage II cultures and various tissues of regenerated stage III *in vitro *plants including leaf (small sections of approximately 25 mm^2^), root (pieces of 0.5–1 mm in length) and pseudostem (handmade cross sections of 1–2 mm in thickness) were also stained histochemically and GUS enzymatic activity (from 200–250 mg tissues) was measured by fluorometry.

## List of abbreviations

ABRE: abscisic acid-responsive element; BLA: baseline luciferase activity; DRE: dehydration-responsive element; ECS: embryogenic cell suspensions; ICEr1: induction of C-repeat/DRE element binding factor expression region 1; I-PCR: inverse-polymerase chain reaction; LT: low temperature; *luc*^+^: codon-optimized firefly luciferase gene; RLU: relative light units; RT-PCR: reverse transcription-polymerase chain reaction; TAIL-PCR: thermal asymmetric interlaced-polymerase chain reaction.

## Authors' contributions

ES performed screenings, cloning of tagged promoters, RT-PCR analysis and participated in sequence analysis as well as drafting the manuscript. SR designed the experiments, participated in the data analysis and screening and finalised the manuscript. ET designed and constructed the tagging vector and participated in sequence analysis. SW maintained cell suspension and tissue cultures, subcultured and regenerated transgenic colonies and participated in screenings. RS made critical revisions and comments, and gave final approval to the manuscript. LS conceived of the study, participated in its design, coordination and analysis, and finalised the manuscript. All authors read and approved the final manuscript.

## Supplementary Material

Additional file 1 **Primer sequences used in this study**. Table to list all primers used for molecular analysis of tagged transgenic banana lines. Click here for file

## References

[B1] Schouten HJ, Krens FA, Jacobsen E (2006). Cisgenic plants are similar to traditionally bred plants: International regulations for genetically modified organisms should be altered to exempt cisgenesis. EMBO Rep.

[B2] André D, Colau D, Schell J, Van Montagu M, Hernalsteens JP (1986). Gene tagging in plants by a T-DNA insertion mutagen that generates APH(3')II-plant gene fusions. Mol Gen Genet.

[B3] Teeri TH, Herrera-Estrella L, Depicker A, Van Montagu M, Palva ET (1986). Identification of plant promoters *in situ *by T-DNA-mediated transcriptional fusions to the *nptII *gene. EMBO J.

[B4] Ow DW, Wood KW, DeLuca M, de Wet JR, Helinski DR, Howell SH (1986). Transient and stable expression of the firefly luciferase gene in plant cells and transgenic plants. Science.

[B5] Van Leeuwen W, Hagendoorn MJM, Ruttink T, van Poecke R, Plas LHW van der, Krol AR van der (2000). The use of the luciferase reporter system for *in planta *gene expression studies. Plant Mol Biol Rep.

[B6] Yamamoto YY, Tsuhara Y, Gohda K, Suzuki K, Matsui M (2003). Gene trapping of the *Arabidopsis *genome with a firefly luciferase reporter. Plant J.

[B7] Alvarado MC, Zsigmond LM, Kovács I, Cséplö Á, Koncz C, Szabados LM (2004). Gene trapping with firefly luciferase in Arabidopsis. Tagging of stress-responsive genes. Plant Physiol.

[B8] Springer PS (2000). Gene traps: tools for plant development and genomics. Plant Cell.

[B9] Mandal A, Sandgren M, Holmström KO, Gallois P, Palva ET (1995). Identification of *Arabidopsis thaliana *sequences responsive to low temperature and abscisic acid by T-DNA tagging and *in vivo *gene fusion. Plant Mol Biol Rep.

[B10] Lee SC, Kim JY, Kim SH, Kim SJ, Lee K, Han SK, Choi HS, Jeong DH, An G, Kim SR (2004). Trapping and characterization of cold-responsive genes from T-DNA tagging lines in rice. Plant Sci.

[B11] Sági L, Remy S, Pérez Hernández JB, Swennen R, Khachatourians GG, McHughen A, Scorza R, Nip WK, Hui YH (2002). Production of transgenic banana (*Musa *species). Transgenic Plants and Crops.

[B12] Sági L, Panis B, Remy S, Schoofs H, De Smet K, Swennen R, Cammue BPA (1995). Genetic transformation of banana and plantain (*Musa *spp.) *via *particle bombardement. Bio/Technol.

[B13] Sági L, Remy S, Verelst B, Panis B, Cammue BPA, Volckaert G, Swennen R (1995). Transient gene expression in transformed banana (*Musa *cv. 'Bluggoe') protoplasts and embryogenic cell suspensions. Euphytica.

[B14] Schenk PM, Sági L, Remans T, Dietzgen RG, Bernard MJ, Graham MW, Manners JM (1999). A promoter from sugarcane bacilliform badnavirus drives transgene expression in banana and other monocot and dicot plants. Plant Mol Biol.

[B15] Dugdale B, Becker DK, Harding RM, Dale JL (2001). Intron-mediated enhancement of the banana bunchy top virus promoter in banana (*Musa *spp.) embryogenic cells and plants. Plant Cell Rep.

[B16] Schenk PM, Remans T, Sági L, Elliott AR, Dietzgen RG, Swennen R, Ebert P, Grof CPL, Manners JM (2001). Promoters for pregenomic RNA of banana streak badnavirus are active for transgene expression in monocot and dicot plants. Plant Mol Biol.

[B17] Remy S, Thiry E, Coemans B, Windelinckx S, Swennen R, Sági L (2005). Improved T-DNA vector for tagging promoters *via *high-throughput luciferase screening. BioTechniques.

[B18] Turner DW, Lahav E (1983). The growth of banana plants in relation to temperature. Aust J Plant Physiol.

[B19] Ganry J (1973). Étude du développement du système foliaire du bananier en fonction de la température. Fruits.

[B20] Israeli Y, Lahav E, Jones DR (2000). Injuries to banana caused by adverse climate and weather. Diseases of Banana, Abacá and Enset.

[B21] Robinson JC (1996). Bananas and Plantains Crop Production Science in Horticulture 5.

[B22] Pérez Hernández JB, Swennen R, Sági L (2006). Number and accuracy of T-DNA insertions in transgenic banana (*Musa *spp.) plants characterized by an improved anchored PCR technique. Transgenic Res.

[B23] Strosse H, Domergue R, Panis B, Escalant J-V, Côte F, Vézina A, Picq C (2003). Banana and plantain embryogenic cell suspensions INIBAP Technical Guidelines 8.

[B24] Topping JF, Agyeman F, Henricot B, Lindsey K (1994). Identification of molecular markers of embryogenesis in *Arabidopsis thaliana *by promoter trapping. Plant J.

[B25] Wei W, Twell D, Lindsey K (1997). A novel nucleic acid helicase gene identified in *Arabidopsis thaliana *by promoter trapping. Plant J.

[B26] Richardson K, Fowler S, Pullen C, Skelton C, Morris B, Putterill J (1998). T-DNA tagging of a flowering-time gene and improved gene transfer by *in planta *transformation of *Arabidopsis*. Aust J Plant Physiol.

[B27] Martirani L, Stiller J, Mirabella R, Alfano F, Lamberti A, Radutoi SE, Iaccarino M, Gresshoff PM, Chiurazzi M (1999). T-DNA tagging of nodulation- and root-related genes in *Lotus japonicus*: expression patterns and potential for promoter trapping and insertional mutagenesis. Mol Plant-Microbe Interact.

[B28] McElroy WD, Seliger HH, McElroy WD, Glass B (1961). Mechanisms of bioluminescent reactions. Light and Life.

[B29] Dickinson R, Francks NP, Lieb WR (1993). Thermodynamics of anesthetic/protein interactions. Temperature studies on firefly luciferase. Biophys J.

[B30] Ueda I, Shinoda F, Kamaya H (1994). Temperature-dependent effects of high pressure on the bioluminescence of firefly luciferase. Biophys J.

[B31] Bade J, van Grinsven E, Custers J, Hoekstra S, Ponstein A (2003). T-DNA tagging in *Brassica napus *as an efficient tool for the isolation of new promoters for selectable marker genes. Plant Mol Biol.

[B32] Mudge SR, Birch RG (1998). T-DNA tagging and characterisation of a novel meristem-specific promoter from tobacco. Aust J Plant Physiol.

[B33] Jeon JS, Lee S, Jung KH, Jun SH, Jeong DH, Lee J, Kim C, Jang S, Yang K, Nam J, An K, Han MJ, Sung RJ, Choi HS, Yu JH, Choi JH, Cho SY, Cha SS, Kim SI, An G (2000). T-DNA insertional mutagenesis for functional genomics in rice. Plant J.

[B34] Alonso JM, Stepanova AN, Leisse TJ, Kim CJ, Chen H, Shinn P, Stevenson DK, Zimmerman J, Barajas P, Cheuk R, Gadrinab C, Heller C, Jeske A, Koesema E, Meyers CC, Parker H, Prednis L, Ansari Y, Choy N, Deen H, Geralt M, Hazari N, Hom E, Karnes M, Mulholland C, Ndubaku R, Schmidt I, Guzman P, Aguilar-Henonin L, Schmid M, Weigel D, Carter DE, Marchand T, Risseeuw E, Brogden D, Zeko A, Crosby WL, Berry CC, Ecker JR (2003). Genome-wide insertional mutagenesis of *Arabidopsis thaliana*. Science.

[B35] Ökrész L, Máthé C, Horváth E, Schell J, Koncz C, Szabados L (1998). T-DNA trapping of a cryptic promoter identifies an ortholog of highly conserved *SNZ *growth arrest response genes in *Arabidopsis*. Plant Sci.

[B36] Mollier P, Hoffman B, Orsel M, Pelletier G (2000). Tagging of a cryptic promoter that confers root-specific *gus *expression in *Arabidopsis thaliana*. Plant Cell Rep.

[B37] Webb KJ, Skøt L, Nicholson MN, Jørgensen B, Mizen S (2000). *Mesorhizobium loti *increases root-specific expression of a calcium-binding protein homologue identified by promoter tagging in *Lotus japonicus*. Lotus japonicus.

[B38] Farrar K, Evans IM, Topping JF, Souter MA, Nielsen JE, Lindsey K (2003). *EXORDIUM *– a gene expressed in proliferating cells and with a role in meristem function, identified by promoter trapping in *Arabidopsis*. Plant J.

[B39] Fobert PR, Labbé H, Cosmopoulos J, Gottlob-McHugh S, Ouellet T, Hattori J, Sunohara G, Iyer VN, Miki BL (1994). T-DNA-tagging of a seed coat-specific cryptic promoter in tobacco. Plant J.

[B40] Plesch G, Kamann E, Mueller-Roeber B (2000). Cloning of regulatory sequences mediating guard-cell-specific gene expression. Gene.

[B41] Sivanandan C, Sujatha TP, Prasad AM, Resminath R, Thakara DR, Bhat SR, Srinivasan R (2005). T-DNA tagging and characterisation of a cryptic root-specific promoter in *Arabidopsis*. Biochim Biophys Acta.

[B42] Remy S, Buyens A, Cammue BPA, Swennen R, Sági L, Galán Saúco V (1998). Production of transgenic banana plants expressing antifungal proteins. I International Symposium on Banana in the Subtropics ISHS Acta Horticulturae.

[B43] Ouellet F, Vazquez-Tello A, Sarhan F (1998). The wheat wcs120 promoter is cold-inducible in both monocotyledonous and dicotyledonous species. FEBS Lett.

[B44] Perales L, Peñarrubia L, Cornejo MJ (2008). Induction of a polyubiquitin gene promoter by dehydration stresses in transformed rice cells. J Plant Physiol.

[B45] Yamaguchi-Shinozaki K, Shinozaki K (2005). Organization of cis-acting regulatory elements in osmotic- and cold-stress-responsive promoters. Trends Plant Sci.

[B46] Yamaguchi-Shinozaki K, Shinozaki K (2006). Transcriptional regulatory networks in cellular responses and tolerance to dehydration and cold stresses. Annu Rev Plant Biol.

[B47] Sherf BA, Wood KV (1994). Firefly luciferase engineered for improved genetic reporting. Promega Notes Mag.

[B48] Dellaporta SL, Wood J, Hicks JB (1983). A plant DNA minipreparation: version II. Plant Mol Biol Rep.

[B49] Aljanabi SM, Martinez I (1997). Universal and rapid salt-extraction of high quality genomic DNA for PCR-based techniques. Nucleic Acids Res.

[B50] Liu YG, Mitsukawa N, Oosumi T, Whittier RF (1995). Efficient isolation and mapping of *Arabidopsis thaliana *T-DNA insert junctions by thermal asymmetric interlaced PCR. Plant J.

[B51] Qin G, Kang D, Dong Y, Shen Y, Zhang L, Deng X, Zhang Y, Li S, Chen N, Niu W, Chen C, Liu P, Chen H, Li J, Ren Y, Gu H, Deng XW, Qu LJ, Chen Z (2003). Obtaining and analysis of flanking sequences from T-DNA transformants of Arabidopsis. Plant Sci.

[B52] Wiame I, Remy S, Swennen R, Sági L (2000). Irreversible DNase I heat inactivation without RNA degradation. BioTechniques.

[B53] Christensen AH, Quail PH (1996). Ubiquitin promoter-based vectors for high-level expression of selectable and/or screenable marker genes in monocotyledonous plants. Transgenic Res.

[B54] Pérez Hernández JB, Remy S, Swennen R, Sági L, Wang K (2006). Banana (Musa sp.). Methods in Molecular Biology Volume 344 Agrobacterium Protocols.

[B55] Jefferson RA, Kavanagh TA, Bevan M (1987). β-glucuronidase as a sensitive and versatile gene-fusion marker in higher plants. EMBO J.

