# Supplementary material for: Characterization and isolation of a T-DNA tagged banana promoter active during in vitro culture and low temperature stress
Source: BMC Plant Biol. 2009 Jun 24;9:77. doi: 10.1186/1471-2229-9-77 (PMC2709630; doi:10.1186/1471-2229-9-77)
Supplement: Additional file 1 — Primer sequences used in this study. Table to list all primers used for molecular analysis of tagged transgenic banana lines. [file 1471-2229-9-77-S1.doc]

**Additional file 1 - Primer sequences**

| Primer | Sequence (5’-3’)* | Application |
| --- | --- | --- |
| AD2 | NGTCGASWGANAWGAA | TAIL-PCR |
| AD2-1 | NTCGTSWGANAWGTT | TAIL-PCR |
| AD2-5 | NGTCGASWCTNAWCAA | TAIL-PCR |
| TAILRBLUC1 | ATAGCTTCTGCCAACCGAAC | TAIL-PCR, I-PCR at RB and RT-PCR |
| TAILRBLUC2 | TCCACCTCGATATGTGCATC | TAIL-PCR, I-PCR at RB and RT-PCR |
| LUCR3 | TCTTCCAGCGGATAGAATGG | TAIL-PCR and I-PCR at RB |
| TAILLBpET2n1 | TTCTTCTGAGCGGGACTCTG | TAIL-PCR and I-PCR at LB |
| TAILLBpET2n2 | GGTTTCGCTCATGTGTTGAG | TAIL-PCR and I-PCR at LB |
| TAILLBpET2n3 | TTAAAAACGTCCGCAATGTG | TAIL-PCR and I-PCR at LB |
| LucL2 | GTGTTGGGCGCGTTATTTAT | I-PCR at RB |
| LucL3 | CTACCGTGGTGTTCGTTTCC | I-PCR at RB |
| LucR5 | GGACTCTGGCACAAAATCGT | I-PCR at LB |
| Luc+R | AGAATCTCACGCAGGCAGTT | I-PCR at LB and cDNA synthesis |
| ActinF3 | CCCAAGGCAAACCGAGAGAAG | RT-PCR forward primer actin |
| ActinR2 | GTGGCTCACACCATCACCAG | RT-PCR reverse primer actin |
| 17-RT-1 | GCCAGGAAACATGACACTTG | RT-PCR forward primer seq. 17-1 |
| 17-RT-2 | TCATACGGCAGCAGGAAAG | RT-PCR forward primer seq. 17-2 |
| 17-RT-3 | ACATGATGTTTCCTAATACATATCTGA | RT-PCR forward primer seq. 17-3 |
| 17-RT-4 | TCCAAGTGCAACATTTAAAACC | PCR and RT-PCR forward primer seq. 17-4 |
| 17-LinkRB-1F | ATCGGAACTCTCATTGATCG | PCR forward primer seq. 17-1 |
| 17-LinkRB-2F | AGAATTCTCATGCGCGTTG | PCR forward primer seq. 17-2 |
| 17-LinkRB-3F | CATGGGAGCAAAGTAAGAGG | PCR forward primer seq. 17-3 |
| 17-LinkLB-1R | AATGCTGGCTTCGCATAGAT | PCR and RT-PCR reverse primer seq. 17-1 |
| 17-LinkLB-2R | ACCACTGTATGGAGCCAAGC | PCR and RT-PCR reverse primer seq. 17-2 |
| 17-LinkLB-3R | AACATTAGCGCTTGCTGTCA | PCR and RT-PCR reverse primer seq. 17-3 |
| 17-LinkLB-4R | TTTGTGAATCCCGTTCTTGTC | PCR and RT-PCR reverse primer seq. 17-4 |
| 17-1F1 | CCGTAGAGATGATCCATGAGC | Cloning promoter 17-1, 1742 and 1354 bp fragment |
| 17-RTLB-1 | TATCGTGATCCCATTTGCTG | RT-PCR forward primer seq. 17-1 |
| 17-RTLB-2 | AGGAAGAACTCCGGTTGAGG | RT-PCR forward primer seq. 17-2 |
| 17-RTLB-3 | TCATGCAATTGATAGGCATC | RT-PCR forward primer seq. 17-3 |
| 17-RTLB-4 | AAACAGCTGCCTATCATGGAG | RT-PCR forward primer seq. 17-4 |
| 17-1R1 | AACACGCGCATAGTCCAAGT | Cloning promoter 17-1, 1742 bp fragment |
| 17-1R2 | AACCCGAGAGAAACGATCAA | Cloning promoter 17-1, 1354 bp fragment |
|  |  |  |

*N refers to A, T, C or G; S refers to C or G; W refers to A or T
